# Development and Use of Gene Therapy Orphan Drugs—Ethical Needs for a Broader Cooperation Between the Pharmaceutical Industry and Society

**DOI:** 10.3389/fmed.2020.608249

**Published:** 2020-12-23

**Authors:** Sandor Kerpel-Fronius, Varvara Baroutsou, Sander Becker, Roberto Carlesi, Luis Collia, Brigitte Franke-Bray, Peter Kleist, Chieko Kurihara, Luis Filipe Laranjeira, Kotone Matsuyama, Shehla Naseem, Johanna Schenk, Honorio Silva

**Affiliations:** ^1^Department of Pharmacology and Pharmacotherapy, Semmelweis University, Budapest, Hungary; ^2^Independent Medical Consultant & Pharmaceutical Medicine Consultant, Athens, Greece; ^3^Consultants in Pharmaceutical Medicine, Dover Heights, NSW, Australia; ^4^Independent Researcher, Bellagio, Italy; ^5^Craveri Pharma, Buenos Aires, Argentina; ^6^Independent Consultant, Basel, Switzerland; ^7^Cantonal Ethics Committee, Zurich, Switzerland; ^8^Quality Assurance and Audit Office, Quantum Medical Science Directorate, National Institutes for Quantum and Radiological Science and Technology, Chiba, Japan; ^9^Eli Lilly & Co., Lisbon, Portugal; ^10^Nippon Medical School, Tokyo, Japan; ^11^Ferozsons Laboratories Ltd., Karachi, Pakistan; ^12^PPH Plus GmbH & Co. KG, Hochheim am Main, Germany; ^13^IFAPP Academy, New York, NY, United States

**Keywords:** gene therapy, rare diseases, orphan drugs, ethics, accelerated approval, health care, spinal muscular atrophy, drug pricing

## Abstract

Gene therapy orphan medicinal products constitute a unique group of new drugs which in case of hereditary diseases are usually administered only once at an early age, in the hope to provide sufficient gene product to last for the entire life of the patients. The combination of an exceptionally large single payment and the life-long clinical follow-up needed for understanding the long-term benefits and safety of gene therapy, represent new types of scientific, financial, social and ethical challenges for the pharmaceutical industry, regulators and society. With special consideration of the uniqueness and importance of gene therapy, the authors propose a three points plan for a close cooperation between the pharmaceutical industry and society to develop orphan gene therapy. (1) In fully transparent health technology negotiations a close and long-lasting, contractually fixed cooperation should be established between the manufacturers and local health-care stakeholders for sharing the medical and scientific benefits, the financial risks as well as the burdens of the post-authorization clinical and regulatory development. (2) The parties should agree on a fair, locally affordable drug price without the usually very high premium price calculated to compensate for the low number of patients. In case of high manufacturing costs, the companies should offer prolonged, 15–20 years long payment by installment with risk-sharing, especially considering that the late outcome of the treatment is unknown. Society should assist scientifically and financially organizing a specific patient registry, treatment in specialized hospitals and adequate long-term follow-up of patients, the coordinated management of financial transactions related to the risk sharing program. (3) The post-authorization treatment and prolonged observation of additional new cases coordinated by society should provide real world data needed for the modern complex regulatory evaluation of gene therapy products by the competent authorities. We assume that fair sharing of the benefits and risks as well as a well-organized cooperation of society with the industry in collecting real world evidence might result in better drug evaluation and improved accessibility due to lower prices. The outlined concept might support gene therapy more efficiently than the presently requested outstandingly high prices.

## Introduction

The Orphan Drug Act accepted in 1983 in the United States of America (US) introduced officially the concept of rare diseases and the corresponding orphan drug designation to stimulate drug development in this area (1). The orphan drug concept subsequently accepted in many countries fulfilled, at least partially, the moral principle that the people suffering from rare diseases should have equal access to treatment and health care regardless of the prevalence of the illness. As a result, drug development for rare diseases has been significantly increased (2–10).

The number of cases defining a rare disease varies throughout different jurisdictions and countries. Expressing the prevalence uniformly per 100,000 inhabitants, the approximate average threshold is between 40 and 50 patients suffering from a particular rare condition (11). Recently, ultra-orphan drugs became accepted as a sub-category of orphan drugs with a cut-off threshold of two patients per 100,000 inhabitants (3, 8, 9, 12).

Due to the rapid advancement of the molecular biological classification of diseases, many new agents targeting small disease entities with well-characterized genetic mutations and pathology will fall into the orphan drug category. The growing abundance of orphan drugs and especially the much higher prices of gene therapies will dramatically increase the financial burden of drug procurement and will leave less money available for the drug treatment of other diseases (4, 8, 9, 13–20). For maintaining the deeply altruistic orphan drug principle, broad scientific, financial and ethical readjustments should be agreed upon by the drug developers and the various health-care organizations considering the special characteristics of orphan gene therapy products. A successful solution must effectively limit their outstandingly high costs while it should also ensure their faster and improving clinical and regulatory development in a broader cooperation with society.

These problems will be discussed using as an example the treatment of spinal muscular atrophy (SMA) with the recently introduced gene therapy product Onasemnogene abeparvovec (Zolgensma®). This case was chosen considering similarities of Zolgensma® with other gene therapy products developed for hereditary diseases, such as once-only treatment, uncertainty of prolonged efficacy and safety and high financial outlay (17–20). Moreover, this high-priced gene therapy received broad attention both in the scientific and public press. Some of the data on pricing and cost-effectiveness of Zolgensma® generated in the US will be used for the discussion due to their abundant and reliable documentation. Based on this example we shall propose a new type of lasting contractual cooperation between the manufacturers and society for sharing the benefits as well as the financial and scientific burdens of the clinical and regulatory development of advanced orphan gene therapy products. We wish to emphasize that our intention is to discuss the general problems related to the pricing of gene therapies rather than to criticize the specific marketing strategy of one medicinal product.

## A Case Study: A Rare Disease Treated With Gene Therapy

### Spinal Muscular Atrophy (SMA)

Spinal muscular atrophy is a genetic disease diagnosed in one in every 10,000–11,000 newborns (21). It has a prevalence of ~1–2/100,000 persons (22). A bi-allelic mutation of survival motor neuron 1 (SMN1) gene causes the disease leading to a deficiency of SMN proteins. There is an additional gene SMN2, that mostly produces rapidly degraded, non-functional SMN molecules due to a splicing error, together with only a small amount of active SMN protein. The severity of the disease depends on the number of SMN2 copies and the amount of the functional SMN protein synthesized (21–23). A lack of SMN protein causes motor neurons to die over time. Other types of neurons, glial cells and blood vessels in the spinal cord and muscles are also affected. In addition, cardiac, gastrointestinal tract and bone abnormalities are frequently present. Therefore, a diagnosis must be made and effective therapy started as early as possible after birth (21).

### Gene Therapy of SMA

In the past, only supportive therapies have been available to alleviate the symptoms of the disease. Recently, two expensive orphan drugs either replacing the defective gene or targeting genetic transcription reached the market. The early studies mostly enrolled patients with severe, rapidly lethal SMA type 1 (23).

Onasemnogene abeparvovec (Zolgensma®) is a complex molecule containing the gene that encodes the SMN protein, together with an enhancer and a promoter needed for proper gene function. The complex construct is linked to a viral vector necessary for carrying the molecule into cells. In genetically impaired neonate mice, a single subcutaneous or intravenous injection of this self-complementary adeno-associated virus serotype 9 construct could deliver enough SMN gene to neurons assuring the adequate further development of the animals for a prolonged time (24, 25). In a phase I study performed on <9-month old patients with SMA type 1, several children showed clinically relevant muscular improvement and most of them did not need further respiratory support. FDA based the early marketing authorization decision on an additional study comparing to historical controls the proportion of patients sitting unassisted for 30 s at 18 months and survival at 14 months of age. For the combined cohorts of 36 children the survival and motor milestone achievements were reported at 24 months (26–28).

In spite of the limited observation period, the once-in-a-lifetime treatment is tentatively assumed to provide a life-long effect. Considering that several factors might influence the duration and extent of the clinical results of gene administration (17), it is scientifically not yet clear whether the injected amount of the gene product and the persistence of its expression will be sufficient over the entire life of the patient. Unfortunately, waning of the therapeutic effects was observed in Hemophilia A patients treated with AAV5-hFVIII-SQ (valoctocogene roxaparvovec). The highest factor 8 activity was observed in the first year, it decreased by 50% in the second year and by additional 10% in the third year. Further follow-up will be needed to understand the dynamic and causes of the waning process (29). A similar waning process occurred 3 years after the intraocular injection of RPE65 gene to patients suffering from Leber's congenital amaurosis. In this case it is speculated that the inserted gene therapy cannot stop the already ongoing degeneration of the photoreceptors at the time of the injection (30).

Zolgensma® received orphan drug designation together with the respective incentives both in the US and the European Union (EU). The U.S. Food and Drug Agency (FDA) granted fast track designation, priority review and a rare pediatric disease priority review voucher (31, 32). Considering the short observations of patients the FDA requested the continued follow-up of the enrolled patients, the entry of new patients in the clinical trial and additional long-term prospective observational studies in their marketing authorization (27, 28). Various countries request similarly long-term follow-up programs. For example, the European Medicines Agency (EMA) gave conditional marketing approval and asked for prolonged monitoring of the enrolled patients together with additional post-authorization efficacy trials with Zolgensma® (33–36).

Nusinersen (Spinraza®), the only other available effective specific treatment for SMA, is an antisense oligonucleotide which binds to the splicing silencer region on the SMN2 pre-mRNA. The modified mRNA is translated into functional SMN protein (37–41). The US list price for intrathecal treatment with Spinraza®, considered in the price calculation of Zolgensma®, is in the first year 750,000 US$ and later 375,000 US$ yearly which would add up to 4.5 million US$ in 10 years (42, 43).

### The Pricing of Zolgensma®

The 2.1 million US$ list-price for the once-only treatment with Zolgensma® became the highest price requested ever for a new medicinal compound (20). For the management of payment, the company suggested an outcome-based, pay-over-time option for a maximum of 5 years, with payment stoppage in case of no observed therapeutic effect (44). Not surprisingly, the high list-price of Zolgensma® led to considerable public controversy. The supporters of this high price argued that it is related to the expenses of developing break-through gene therapies, to the price of the prolonged, chronic, supportive care needed by the children suffering from SMA which is about 4.1 million US$ per 10 years. Furthermore, the price is below the cost of treating ultra-rare genetic pediatric diseases (<2 patients/100,000 inhabitants) (3, 12), which is, on average, around 4.4–5.7 million US$ over 10 years (44, 45). The price of one Quality-Adjusted Life Year (QALY) gained for treating SMA type 1 in children <8 months was calculated to be 243,000–248,000 US$ by the Institute of Clinical and Economic Review (ICER) (42, 46). The New England Comparative Effectiveness Public Advisory Council concluded that to reach the generally cited cost-effectiveness thresholds of 50,000–150,000 US$ per QALY gained, the price should be decreased to around 310,000–900,000 US$ per treatment. In the case of similar thresholds for Life-Years Gained, the appropriate price would be 710,000–1.5 million US$ (43). These calculations were originally based on assumed price, but are nevertheless acceptable since the final list price became almost identical. The company also related the high price of Zolgensma® to the 10-year long, 4.5 million US$ calculated summary treatment cost of Spinraza®. It is important to note that the prices of Spinraza® result in cost-effectiveness ratios higher than those accepted in the US and Europe, respectively (47, 48). Nevertheless, the drug became reimbursed in most of the European countries indicating the strong effect of public pressure on reimbursement policy. It is important to point out that the results of the pharmacoeconomic calculations are based on vague assumptions since nobody knows how long the effect of gene or translation modifiers will last. Finally, it should be emphasized that there is a significant difference in the payment constructions of Spinraza® and Zolgensma®. In the first case, payment is directly related to the continuation of the therapy, while in case of once in a life-time gene therapy the patients have to pay up-front the entire cost without knowing how long the effect will last. The present 5 years long outcome-based, pay-over-time option policy covers only early ineffectivity, a possible later waning of the effect is not considered.

In this paper, only the US prices are analyzed, because in the USA the companies themselves determine the list price. The list price will be usually modified and specific rebates will be agreed upon in confidential negotiations with the various payers in the US. Nevertheless, the outstandingly high price difference between the list prices of Zolgensma® and other medicinal products will most probably remain similar in other countries and will exert an exceptionally high impact on the local drug budgets. For example, in Germany a price of 1,945,000 EUR, close to the US price was announced, which will be renegotiated based on the local cost-effectiveness evaluation after 1 year. In other EU Member States, the negotiations are ongoing (49). The comparatively outstanding high list price of gene therapy alarms the public worldwide.

### Clinical, Financial, and Ethical Issues Related to the Accelerated Marketing Authorization of Orphan Gene Therapies

Following the above short presentation of the development, marketing authorization and pricing of Zolgensma®, let us analyze what we can learn from this experience for improving both the affordability and post-marketing evaluation of gene therapy products. The need of higher priced orphan drugs for small patient populations suffering from rare diseases is now ethically broadly accepted. This led to the orphan drug legislation with the provision of economic incentives to the pharmaceutical companies for supporting drug development for rare diseases. Regulatory agencies devised several further means for making life-saving drugs available to the patients as early as possible after the confirmation of basic efficacy and safety parameters (1, 31–35). As described above, Zolgensma® was approved based on a single trial with few patients and short follow-up. Although rapid approval opened the possibility to buy the drug for seriously sick patients, unfortunately, the available data did not provide adequate clinical information on the long-term therapeutic benefit and safety of Zolgensma® gene therapy. A considerable part of the scientific work addressing the long-term issues is shifted to the post-authorization phase (50). Therefore, the FDA obliged Novartis to follow up the patients enrolled into the registration trials for 5 years annually and later by phone calls for further 10 years. In addition, a prospective multicenter, multinational long-term observational voluntary registry should be organized following patients suffering from all types of SMA for 15 years (27, 28). The sponsor should enroll 500 patients into this voluntary observational project. The EMA prescribed two additional studies in its conditional approval, one in patients with SMA type I older than 6 months and another trial in children younger than 6 months with genetically confirmed SMA (36). These requirements assure the continuous participation of the pharmaceutical industry representing a heavy burden for the industry in the further evaluation of the drug.

It is also obvious that a considerable part of the required post-authorization clinical observations has to be performed by society. The many additional cases and the very long follow-up time needed for proving the long-lasting effectivity and safety of gene therapy place exceptionally high burden also on the health-care system. Most of the data provided by society will fall into the category of real world evidence feeding the above-mentioned voluntary registry of Zolgensma® treated patients. The coherent collection and the combination of data emerging from classical clinical trials and real world data is becoming an increasingly important approach for the regulators to offer a well-balanced evaluation of orphan products as suggested by Eichler et al. (51). Much organizational and financial effort is needed from society to provide reliable, easily evaluable real world data. Unfortunately, the extremely high price of orphan gene therapy is counter-productive in this respect, since it reduces the number of treated patients. The introduction of high premium prices for orphan drugs was made possible by marketing elements included in the altruistic orphan drug concept. Especially the price claims to compensate for the expected low sales volume needed to cover the needs of the small patient population suffering from rare diseases and calculating the losses due to unsuccessful development programs might jointly lead to an highly inflated industrial profit (7, 16, 52–54). Similar to many other orphan drugs, the very high price of Zolgensma® was justified by the severity of the condition and the short life expectancy of the patients (8, 9, 42, 43). The breakthrough nature and the possible life-long effect of gene therapy served as great added benefits in the price calculation. Naturally, the premium price claim for Zolgensma® must take also into consideration how much the patient's families and society are willing and can pay for the expected long-lasting therapeutic results in the various countries. Interestingly, the rarity of a disease seems to be of low importance for the public acceptance of high prices of orphan drugs (55).

The construction and manufacturing of the gene therapy medicinal products are material factors determining their exceptionally high prices. In addition, the overall large expenses of broad prior research leading to the development of high-tech products are often considered in the price calculation and are frequently cited for explaining their exaggerated prices. However, this is a questionable argument and it should be judged critically, since a large part of previous basic research used was probably covered by public funds and its results are available from the scientific literature (54). In addition, it is usually impossible to calculate the exact value of the public scientific contribution and relate it quantitatively to a given gene therapy. Proven research contribution by society to the development of a commercialized product should be principally deleted from price calculation. For example in the case of Zolgensma® the early development was supported by the National Institute of Health with more than 450 million US$ in grants citing “spinal muscular atrophy.” Several large US charity organizations provided additional large grants (52). Only the documented research and development contributions to a given product of the industry possibly together with expenses of closely related research but not leading to commercialized product should be included in the determination of the price.

Health insurance companies around the world find it hard to incorporate the very highly priced gene therapies into their policy when the long-term outcome is not yet proven. Besides the high budget impact, they consider also the effect of the disease on the patients and caregivers (8, 9). The high price decreases the number of treated patients and additionally dramatically increases the inequality of access to health care since it selectively affects patients from low-income populations (56, 57). Taking into account all these economic and social factors some insurers negotiate subscription-based contracts in which a lump-sum payment, permitting the unlimited access of patients within a defined period, is made (20). Others try to make use of risk-sharing programs offered either as financial or performance/outcome models. Unfortunately, risk-sharing is burdened by high administrative costs, lack of transparency associated with conflict of interest, and uncertainty whether the costs will be paid (45, 58, 59). Novartis offers a risk-sharing plan for Zolgensma® to be paid in 5 years with a possibility to stop payment if the therapy proves to be ineffective (44).

## Discussion

A frequently asked question is how far society can cover the upward pricing spiral elicited by new types of drugs, primarily by gene therapy products. In the US, ICER convened a special expert meeting for discussing evidence generation, assessing pricing, value, and affordability of gene therapy (8, 9, 59, 60). Meanwhile, the generally over-optimistic hope of the parents led to surprising approaches to secure Zolgensma® for their seriously sick children in countries where the drug was not yet marketed. For example, in Hungary and Slovakia following intensive Internet campaigns, the full treatment costs for five children were covered from public donations. The patients paid the full price and received treatments in a Hungarian hospital where the staff was trained by the pharmaceutical company to administer Zolgensma®. According to Internet communications, the children responded to the therapy in a way similar to those described in the literature (61). Subsequently, Novartis offered to make the gene therapy available free of charge to 100 children selected by lottery in the EU where the drug, at that time, was not yet registered. In both situations, regulatory authorities gave consent for administering the drug (62). Presently, following EMA marketing authorizations, price agreements are negotiated in various European countries (56, 57).

However, such non-official acquisition strategies are too fragile to provide equitable access for all the patients needing expensive orphan drug treatments. Indeed, there is a real possibility that due to the intensive pressure of few patients for obtaining disproportionally expensive treatments, an increasing fraction of the local drug budgets might be channeled away from the majority of patients suffering from more frequent diseases, thereby increasing the health-care burden of other patients. The more likely possibility is that without establishing a properly balanced price band for gene therapy products, the present drug reimbursement system will not be able to cover gene therapy distorting further the equity of access to required drugs (16). International overviews already show that the proportion of patients treated with expensive orphan drugs drops with decreasing national income (56, 57).

### The Need for an Improved Sharing of Benefits and Risks of Gene Therapy Based on Long-Term Scientific, Financial Cooperation

The above discussed considerations led us to envisage a more sophisticated solution involving both the pharmaceutical industry and several healthcare-related organizations participating in the clinical development and use of gene therapies. We propose that due to the special clinical pharmacological properties of orphan gene products and their exorbitantly high price the pharmaceutical industry should be persuaded by society to accept a more generous benefits and risks sharing program to improve the access of patients to the drug. On the other side, the public should provide much more effective support for the investigation of the effectivitiy of gene products. The aim should be established by a goal-oriented long-term contractual cooperation between the parties.

The concept of benefit sharing is essentially a “gesture of solidarity,” meaning procedural and distributive justice in drug research stipulating the ethical obligation that some of the advantages gained by a sponsor should be shared with the subjects and communities participating in the project. Unfortunately, benefit sharing in clinical research is not enshrined into a binding legal system. Benefit sharing is frequently described in relation to international clinical drug development performed by capital strong pharmaceutical companies in developing countries (63). It can take many forms, for example providing the investigated drugs, diagnostic methods or complex therapies at a decreased price to the local community. Ballantyne (64) suggests that a global tax on international research carried out in developing countries should be collected from sponsors for providing fair benefits. The greatest ever benefit-sharing, public-private partnership project is presently underway for managing cooperative research and world-wide access to vaccines against COVID-19 infection (65). Considering the gesture of solidarity, one might argue that many patients entering Zolgensma® gene therapy trials were underprivileged members of the population living in the US and in some other wealthy countries. Many members of the society are underprivileged in the sense that the price of Zolgensma® is 33 times the per capita income in the US and a large section of the population is not covered at all or has only limited health insurance policy not suitable for buying high priced marketed gene therapy (52). The situation will be similar in many other countries in the world. We suggest that abandoning the additional high premium price calculated for compensating the small patient number would be an appropriate benefit-sharing method for orphan gene products.

Benefit sharing should be accompanied by a risk-sharing agreement covering 15–20 years which is in line with the clinical observation period prescribed by the FDA for recording long-term effects (27, 28). Therefore, the risk-sharing period should be much longer than 5 years offered by the company (44). A reliable risk-sharing agreement must be based on a robust evidence of drug performance, including well-defined clinical parameters used for the evaluation of the therapeutic and side effects and finally on a very careful follow-up of many patients (66–68). Unfortunately, in the case of gene therapy orphan drugs these parameters frequently cannot be well-characterized. Since the long-term clinical outcome of gene therapy is not yet known, a robust health technology assessment analysis becomes problematic. The evaluation of cost-effectiveness, such as the incremental cost-effectiveness ratio, the quality adjusted life years (QALY) calculations are based on imprecise estimations. Therefore, such calculations should be combined with a budget impact analysis, which provides jointly with the above calculations a more reliable background for understanding the effect of introducing an expensive new drug into the health care system. Finally, it should be realized that the conclusions of health technology assessments are usually interwoven with many value judgments and societal considerations, essentially they represent a combination of financial and ethical views (69). On this complex basis, health technology experts can work out a relatively fair estimate of a helpful risk-sharing agreement for gene therapy.

Beside the benefit- and risk-sharing programs offered by the pharmaceutical companies society must also add its share to effective post-authorization activities primarily in the organization and support of an added real-world evidence program. The rapid improvement of advanced therapy products provides a moving target for the real world follow-up. The observations should be carried out in specialized medical centers able to provide scientifically high-level data extending those derived from the earlier small, frequently single-arm regulatory clinical trials. These centers should also scientifically contribute to the continuous development of these new type of products targeting only small patient groups (51). For such complex post-launch activities health technology assessment committees, payers, patient support groups, the general public as well as the clinical researchers, hospitals and research foundations have to cooperate very closely. The aim should be to plan and coordinate precisely the financial as well as the scientific commitments of both the pharmaceutical company and health-care stakeholders. This should include the buying of drugs by payers for treating a given number of additional patients, providing treatment and data management costs from local resources for the required high-quality real world observational programs. Such a strategy is in line with the recently endorsed ethical recommendation for community engagement in human research by the Council for International Organizations of Medical Sciences (CIOMS) (70) and publications discussing advantages of public involvement (71–73).

The pharmaceutical industry is also aware of the need for a well-organized post-authorization cooperation with the society. At a special expert meeting dealing with the affordability of gene therapy, they formulated very similar recommendations (59). The pharmaceutical industry considers the organization of a robust patient registry especially important, advocates outcome payment options and various loan, reinsurance, manufacturer or government financing options for covering the high drug price. The industry experts argue that for this purpose, the drug producer must provide a completely transparent explanatory documentation proving the need for high price. They advocate a close cooperation with health technology assessment groups, which should lead to a fair price acceptable for both the payers and patient groups. Characteristically, special price reduction for gene therapy is not a recommended option by the industry. Finally, they propose that negotiations with the payers should be started early, preferably before the authorization of a drug.

The optimal organizations for negotiating fair financial agreements and efficient cooperation between the pharmaceutical industry and society are the health technology assessment groups. It is now broadly accepted that they cannot deal exclusively with financial aspects, they must consider also ethical and societal values such as good quality care, equity and solidarity as formulated for example in the value statement of EUnetHTA (74). The special case of gene therapy offering the possibility of life-long effect following one intervention in a lethal disease sparked considerable discussion regarding the nature of a fair price. Some argue along the line what would patients and society willing to pay for influencing all or several problems associated with a given disease, such as the severity, risk protection, equity of diseases, caregiver burden, financial losses, etc. According to this view in catastrophic health situation such as lethal hereditary diseases the value-creating elements should essentially justify much higher cost-effectiveness thresholds than used generally for orphan drugs (75). However, others argue that considering ethical, political, sociological principles society should enforce as much distributive equivalence as possible when allocating resources for advanced therapies (76).

### Three Important Pillars of the Recommended Contractual Agreement

1. These divergent financial and ethical views must be weighted when health technology assessment groups make price and reimbursement decisions. High cost-effectiveness thresholds will decrease the number of patients treated and further use will be self-limiting due to a substantial increase of the financial burden for the society. Our principally different approach advocating the decrease of the premium price with parallel increase of the real-life research contribution by society might be quite advantageous for the pharmaceutical industry due to an increased number of patients involved and improved evaluation of the real world effectivity. Based on real-life evidence an outcome related payment procedure could be also established in which the price would be adjusted stepwise according to the maturity of the experience. The most important condition for the negotiated contractual approach is to make available a completely transparent scientific and financial development documentation by the industry as well as by the society covering their real life research contribution. On this basis, the parties could agree on a fairer price considering the proven research, development, production and marketing costs without calculating extra premium since the company gets financially and scientifically valuable real world research support as a compensation. Such evaluation needs the expertise of many different specialists. Health technology evaluations are presently done at national or even at lower levels of society resulting in qualitatively quite different decisions. Reaching the market is prolonged and regionally fragmented making the organization of rational, broad, cooperative gene therapy programs difficult. Therefore, we believe that such complex evaluation should be done internationally. Fortunately, we have now several international health technology organizations such as the INAHTA, EUnetHTA, RedETSA, HTAsiaLink, WHO-HTA cooperations covering different regions (77). These organizations would be in the best position to make regional analyses, scientific and financial recommendations before the final marketing of the new products. Following the subsidiarity principle of the EU only minor adjustments would be necessary at the national levels after marketing authorization for adapting the joint recommendations to the local circumstances and fine tuning the cooperation for collecting real world data.
2. The usually expensive production of advanced orphan gene medicinal products will keep the cost high even without the added premium price. Society has to make difficult decisions as to how much of the available healthcare resources can be made available for treating patients with gene therapy. Purchasing these expensive medicinal products will frequently necessitate accruing additional grants from private and public research funds. Besides making these decisions, society should help to organize and manage disease specific groups for registering and following the patients. These organizations should also be responsible for collecting the data on patients for managing drug procurement and payments according to the risk-sharing program. The combination of all these organizational and financial tasks in special funds are of great practical importance for supporting the high-level scientific evaluation of all the treated patients and assuring a reliable long-term financial partnership with the sponsor (60). Considering the many years of follow-up needed for evaluating gene therapy, an installment payment and risk-sharing agreement running for 15–20 years could be the most appropriate time frame for easing the financial burden. Although these considerations could be helpful for managing other orphan drugs, we believe that these specific and quite cumbersome recommendations would be warranted only for gene therapies which need a very prolonged follow-up after a single treatment and large up-front payment.
3. The last important pillar of the long-term agreement should be based on the close cooperation of the patient groups with clinical researchers and hospitals. In this model, the patient groups would play a significant organizational role in the research programs especially during the post-authorization, real-world experience based development. This cooperation should be associated with an educational program teaching patient groups to represent people suffering from the same disease considering also the impact on the wider society. It is important that these patient organizations should be self-supported and independent of industrial economic influences (78). We believe that it would be wise to link closely the national market entry of gene products with the organization of the suggested broad health-care and financial cooperation to optimize the patient-oriented drug supply and the efficient evaluation of treatment data. In countries with a single state-controlled insurance system the organization of the suggested cooperation might be simpler. In other countries with several, frequently competing health insurance companies the easiest approach would be to form a coalition to provide a solid background for cooperating with the drug producer.

Summing up, we are convinced that for firmly anchoring gene therapies in the medical practice fundamental changes are needed in their pricing and regulatory evaluation. According to our opinion, the further increase of cost-effectiveness threshold leading to a higher price could support development at the producer side, but poor affordability would significantly hamper the broad clinical use of gene therapy. We suggest that close financial, regulatory and clinical cooperation between the pharmaceutical industry and society could decrease significantly the price by generous benefit and risk sharing offered by the industry coupled to compensating gestures by society. The latter should assume responsibility for the medical and financial organization of a thorough real-life evaluation of gene therapy on a large patient population needed for the modern, complex regulatory evaluation. We hope that these ideas will promote further debate among all stakeholders to support broad access to gene therapy in the future.

## Data Availability Statement

The raw data supporting the conclusions of this article will be made available by the authors, without undue reservation.

## Author Contributions

SK-F initiated this work and wrote the manuscript in close scientific cooperation, consultation, and discussion with the co-authors. All authors are members of the IFAPP Ethics Working Group. All authors contributed to the article and approved the submitted version.

## Conflict of Interest

LL was employed by Eli Lilly & Co. LC was employed by Craveri Pharma. SN was employed by Ferozsons Laboratories Ltd. JS was employed by PPH plus GmbH & Co. KG. BF-B is a consultant affiliated with IFAPP and IFAPP Academy. The remaining authors declare that the research was conducted in the absence of any commercial or financial relationships that could be construed as a potential conflict of interest.
